# Digital PCR Discriminates between SARS-CoV-2 Omicron Variants and Immune Escape Mutations

**DOI:** 10.1128/spectrum.05258-22

**Published:** 2023-06-12

**Authors:** Steven C. Holland, LaRinda A. Holland, Matthew F. Smith, Mihyun B. Lee, James C. Hu, Efrem S. Lim

**Affiliations:** a Center for Fundamental and Applied Microbiomics, Biodesign Institute, Arizona State University, Tempe, Arizona, USA; b School of Life Sciences, Arizona State University, Tempe, Arizona, USA; University of Utah and ARUP Laboratories

**Keywords:** SARS-CoV-2, digital PCR, monoclonal antibody, immune escape mutations, clinical diagnostics

## Abstract

As severe acute respiratory syndrome coronavirus 2 (SARS-CoV-2) continues to evolve, mutations arise that will allow the virus to evade immune defenses and therapeutics. Assays that can identify these mutations can be used to guide personalized patient treatment plans. Digital PCR (dPCR) is a fast and reliable complement to whole-genome sequencing that can be used to discriminate single nucleotide polymorphisms (SNPs) in template molecules. Here, we developed a panel of SARS-CoV-2 dPCR assays and demonstrate its applications for typing variant lineages and therapeutic monoclonal antibody resistance. We first designed multiplexed dPCR assays for SNPs located at residue 3395 in the *orf1ab* gene that differentiate the Delta, Omicron BA.1, and Omicron BA.2 lineages. We demonstrate their effectiveness on 596 clinical saliva specimens that were sequence verified using Illumina whole-genome sequencing. Next, we developed dPCR assays for spike mutations R346T, K444T, N460K, F486V, and F486S, which are associated with host immune evasion and reduced therapeutic monoclonal antibody efficacy. We demonstrate that these assays can be run individually or multiplexed to detect the presence of up to 4 SNPs in a single assay. We perform these dPCR assays on 81 clinical saliva SARS-CoV-2-positive specimens and properly identify mutations in Omicron subvariants BA.2.75.2, BM.1.1, BN.1, BF.7, BQ.1, BQ.1.1, and XBB. Thus, dPCR could serve as a useful tool to determine if clinical specimens contain therapeutically relevant mutations and inform patient treatment.

**IMPORTANCE** Spike mutations in the SARS-CoV-2 genome confer resistance to therapeutic monoclonal antibodies. Authorization for treatment options is typically guided by general trends of variant prevalence. For example, bebtelovimab is no longer authorized for emergency use in the United States due to the increased prevalence of antibody-resistant BQ.1, BQ.1.1, and XBB Omicron subvariants. However, this blanket approach limits access to life-saving treatment options to patients who are otherwise infected with susceptible variants. Digital PCR assays targeting specific mutations can complement whole-genome sequencing approaches to genotype the virus. In this study, we demonstrate the proof of concept that dPCR can be used to type lineage defining and monoclonal antibody resistance-associated mutations in saliva specimens. These findings show that digital PCR could be used as a personalized diagnostic tool to guide individual patient treatment.

## INTRODUCTION

The evolution of severe acute respiratory syndrome coronavirus 2 (SARS-CoV-2) brings challenges to disease epidemiology and patient treatment. Genomic mutations arise that define phylogenetic lineages, alter virus properties, and have functional consequences of clinical significance (1). In early November 2021, the SARS-CoV-2 Delta variant and its sublineages were the predominantly circulating lineages in the United States (2). On 26 November 2021, the World Health Organization designated the SARS-CoV-2 Omicron variant as a variant of concern (VOC) (3). Genome sequencing showed that the Omicron lineages contained approximately 50 unique mutations compared to previous variants of concern (4). Phylogenetic analysis indicated that the Omicron lineages arose independent of the Delta lineage, likely from a prolonged infection in an immunocompromised patient or an animal host (5). The Omicron BA.1 lineage eventually rose to be the dominant circulating lineage, displacing Delta lineages at a rate faster than previous variants (6). This increased rate of displacement is likely due to increased immune evasion and infectivity of the early Omicron BA.1 and BA.2 variants (7–9).

Since their initial emergence, Omicron lineages have diversified, with multiple sublineages branching from the ancestral BA.1 and BA.2 lineages. Currently circulating variants (e.g., BA.2.75.2 and BQ.1.1) are resistant to neutralization by sera from vaccinated individuals and from vaccinated individuals who recovered from an early Omicron breakthrough infection (BA.1, BA.2, or BA.5) (10). Worryingly, circulating sublineages have also independently evolved mutations within the receptor binding domain (RBD) of the spike protein that escape neutralization by the therapeutic monoclonal antibodies (MAbs) bebtelovimab, bamlanivimab, etesevimab, casirivimab, tixagevimab, and others (10–14; reviewed in reference 15). The R346, K444, L452, N460, E484, and F486 residues may be particularly important in MAb resistance and SARS-CoV-2 pathology (16). Due to the limited methods to determine if a patient is infected with a resistant variant, the FDA issues blanket guidance to withdraw emergency use authorization (EUA) for MAbs based on general variant presence (17). This significantly limits patient access to life-saving treatment options regardless of what variant they are infected with (e.g., cocirculating variants that are susceptible to MAbs). Hence, methods to rapidly determine if SARS-CoV-2-positive patient specimens harbor resistance mutations are of critical importance in guiding therapeutic treatment.

Whole-genome sequencing is the most comprehensive method for genotyping SARS-CoV-2. However, the cost, time requirements, and required technical expertise leave a need for complementary methods to identify variants of concern and mutations of interest in clinical specimens. Digital PCR (dPCR) has emerged as a technology that can be used to detect and differentiate single nucleotide polymorphisms (SNPs) in template DNA (18). In the QuantStudio Absolute Q Digital PCR system (Thermo Fisher, MA, USA), template DNA is partitioned into over 20,480 microchambers. Within each microchamber, an endpoint PCR is performed which contains primers and probes specific to template DNA. After the PCR assay has been completed, fluorescence intensity is then used to determine whether a microchamber is “positive” or “negative” for each probe target, and the number of positive microchambers can be used to calculate template presence (19). The robustness and sensitivity of dPCR to detect template differences of only a single nucleotide have made it useful in practical applications of viral identification and quantitation, determination of allelic imbalance, and wastewater viral variant surveillance (20–22).

In this study, we demonstrate the usefulness of dPCR in detecting polymorphisms in SARS-CoV-2 genomes obtained from clinical saliva specimens. We first develop a dPCR assay that can be used to discriminate between the Delta, Omicron BA.1, and Omicron BA.2 lineages. We verify the effectiveness of the assay by determining lineage designations for 596 SARS-CoV-2-positive clinical saliva samples and verify those determinations by Illumina next-generation sequencing (NGS). We then demonstrate the ability of dPCR to identify mutations in up to 4 genomic loci in a single reaction vessel and use this ability to detect SNPs associated with immune escape in 81 clinical saliva samples, which are confirmed by whole-genome sequencing.

## RESULTS

### Digital PCR assay to distinguish Delta, Omicron BA.1, and Omicron BA.2 utilizing two SNPs in the *orf1ab* gene.

**FIG 1:**
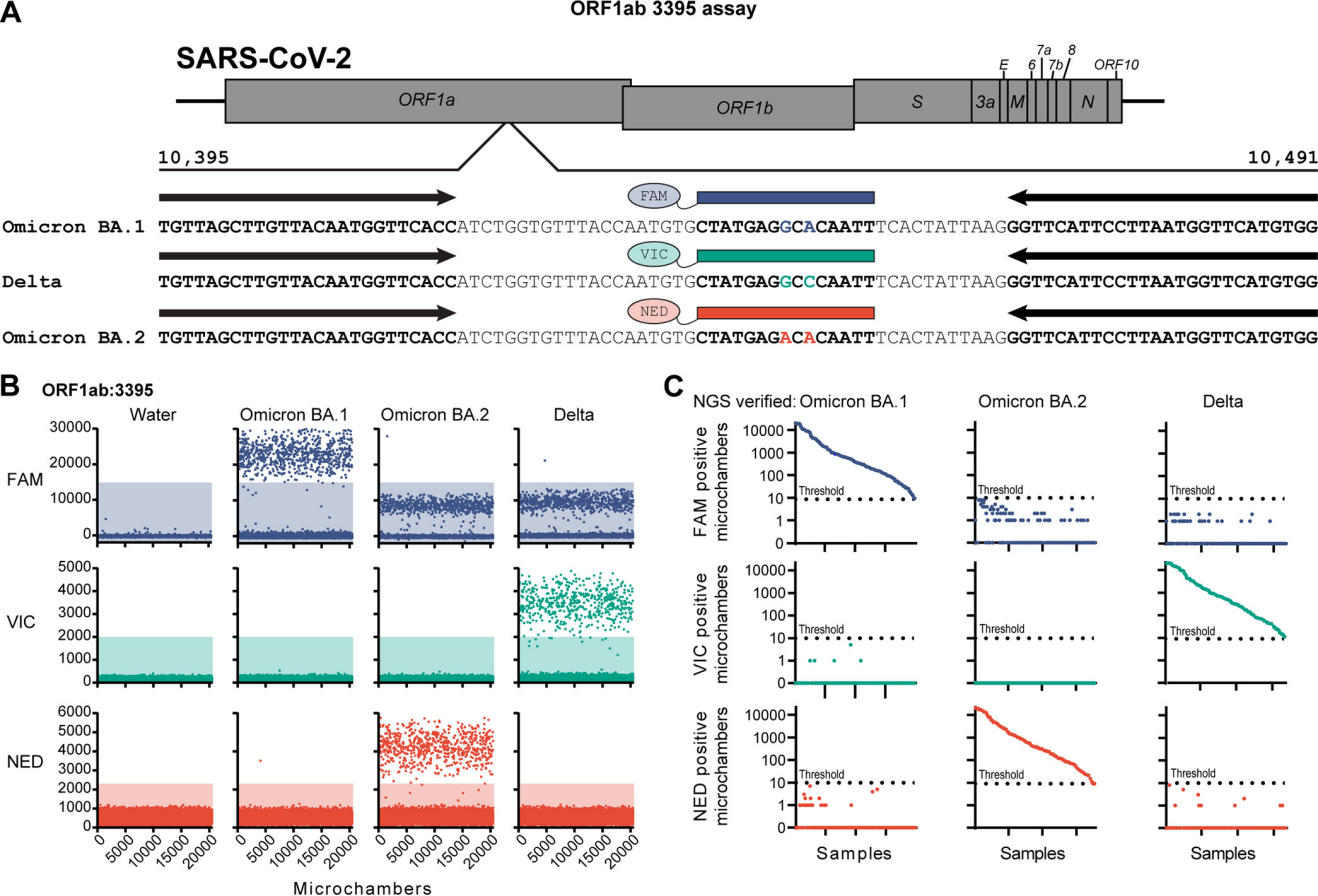
Digital PCR assay for the determination of the Delta, Omicron BA.1, and Omicron BA.2 lineages. (A) Schematic showing annealing locations of primers (black arrows) and probes (colored boxes) on the SARS-CoV-2 genome. (B) Representative fluorescence intensities of dPCR microchambers for synthetic DNA constructs. Positive microchambers are those exceeding fluorescence thresholds (shaded regions). (C) Number of positive microchambers (maximum number of chambers is 20,480) resulting from each saliva sample. Samples are grouped by Illumina sequencing lineage determination and sorted by positive microchamber count of the respective lineage-specific probe. Dotted line indicates the positive threshold value (9 microchambers).

Since the effectiveness of therapeutic treatments can differ between SARS-CoV-2 lineages, a set of dPCR probes was designed to discriminate between the Delta and Omicron BA.1 and BA.2 lineages using SNPs found in the *orf1ab* gene (Fig. 1A). The probes hybridize at nucleotides 10440 to 10454 and are used to detect substitutions found in the Delta and Omicron lineages located at amino acids R3394 and P3395. The probe homologous to the Delta lineage sequence shares the same nucleotide sequence as the Wuhan-Hu-1 reference sequence over the probe region. The Omicron BA.1 probe discriminates between the Delta sequence at the c10449a nucleotide substitution (amino acid P3395H). The Omicron BA.2 probe discriminates between the Delta sequence at the c10449a (amino acid P3395H) and g10447a (synonymous mutation in R3394) nucleotide substitutions. The Delta and Omicron BA.1 and BA.2 probes have either a FAM, VIC, or NED fluorescent dye, respectively, connected to the 5′ end of the oligonucleotide and a minor groove binder (MGB) moiety on the 3′ end.

The efficacy and specificity of probes were tested using synthetic DNA constructs (Fig. 1B). The Delta (VIC), Omicron BA.1 (FAM), and Omicron BA.2 (NED) probes showed a clear fluorescence response when assayed with their homologous templates. The Omicron BA.1 probe also showed a fluorescence response to the Delta and Omicron BA.2 templates. This signal was lower in fluorescence intensity than those observed from Omicron BA.1 templates, but it was higher than the negative signal observed in reaction mixtures containing no template DNA. There was a negligible response of the Delta (VIC) and Omicron BA.2 (NED) probes when used on their mismatching templates. Fluorescence threshold values for each probe were set to exclude the range of negative values found in mismatching templates. The fluorescence threshold for Omicron BA.1 (FAM) was set above the nonspecific fluorescence values observed in the mismatching template assays.

In order to determine the effectiveness of the *orf1ab* dPCR assay on clinical specimens, we performed the 3-probe multiplex dPCR assay and Illumina next-generation sequencing on 596 SARS-CoV-2-positive saliva samples (188 Delta, 215 Omicron BA.1, 193 Omicron BA.2). To receive a positive result for a particular probe, the sample required 9 or more microchambers to have a fluorescence intensity above the threshold value. During the dPCR assay scoring, Illumina sample lineages were blinded to scorers. Using these criteria, 540 samples tested positive for a single lineage using the dPCR assay. All 540 samples had lineage designations by dPCR concordant with Illumina whole-genome sequencing designations. No samples displayed a positive response from probes discordant with the genome sequencing designations (Fig. 1C; also see Data Set S1 in the supplemental material). There were 56 samples in which no probe passed the positive chamber count. Analysis of genome sequences revealed no sequence mismatches in the primer and probe binding regions. TaqPath *Orf1ab* gene *C_T_* values of these negative samples were found to be significantly higher than samples with positive outcomes, indicating that viral load may contribute to assay efficacy (positive samples’ median *C_T_*, 22.05, negative samples’ median *C_T_*, 24.34, *P < *0.0001; Mann-Whitney test). Together, this demonstrates that dPCR can use two nucleotide polymorphisms to discriminate between the Delta, Omicron BA.1, and Omicron BA.2 lineages in saliva specimens.

To further illustrate the ability of dPCR to discriminate between SARS-CoV-2 lineages, a second assay was developed (Fig. S2). This assay used a nine-nucleotide deletion in the spike gene (g21987-t21995) found in the Omicron BA.1 lineage, but not the Delta or Omicron BA.2 lineages. The performance of this assay was similar to the Orf1ab assay, successfully discriminating Omicron BA.1 from Delta and Omicron BA.2 (Data Set S2). Together with the Orf1ab assay, these assays demonstrate the ability of dPCR to perform lineage subtyping on SARS-CoV-2 genomes in clinical saliva samples.

### Spike RBD mutations associated with immune escape are increasing in abundance.

**FIG 2:**
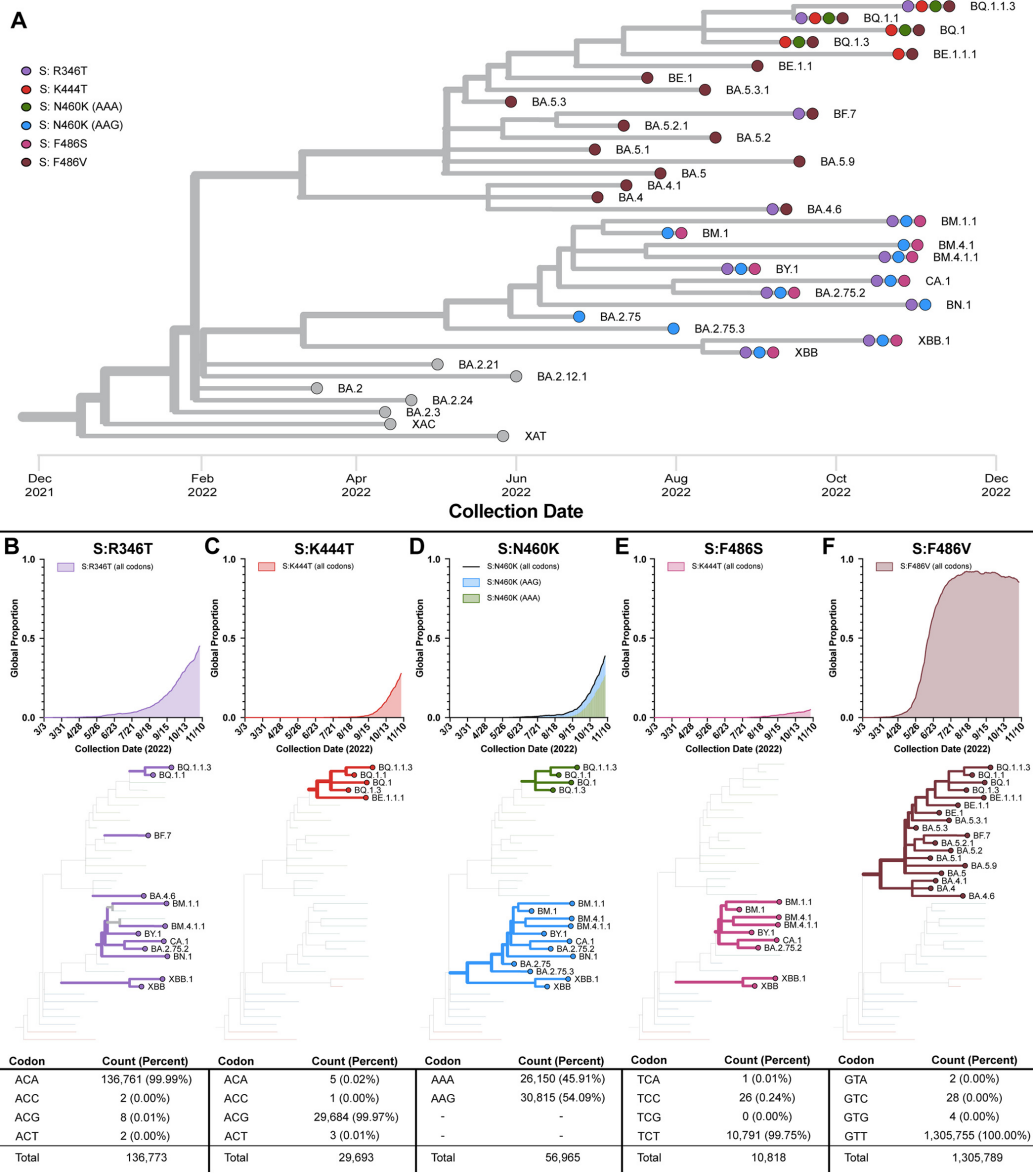
Phylogenetic distribution of mutations that evade therapeutic monoclonal antibodies. (A) Phylogenetic tree of representative circulating sublineages. Colored circles indicate the presence of a spike RBD mutation found in at least 75% of sequences within that lineage. (B and F) Global frequency, phylogenetic distribution, and codon frequencies for R346T (B), K444T (C), N460K (D), F486S (E), and F486V (F) mutations.

Another potential diagnostic application is in discerning the presence of amino acid mutations that may render therapeutic monoclonal antibodies ineffective. To observe the frequency of spike RBD mutations that provide immune escape properties, the GISAID database was queried for SARS-CoV-2 Omicron sequences containing five mutations associated with immune escape, R346T, K444T, N460K, F486V, and F486S (10) (Fig. 2). The L452R mutation within the spike RBD region has also been strongly associated with immune escape (10) but has been the subject of reverse transcriptase quantitative PCR (RT-qPCR) detection (23), so it was omitted from analysis. We found that many of these mutations are found distributed across multiple sublineages (Fig. 2A). We observed that the R346T mutation has been rising in frequency since May 2022 and has arisen in the BA.2, BA.4, and BA.5 lineages as well as in the XBB recombinant lineages (Fig. 2B). The K444T mutation has been rising in global frequency since September 2022, arising within the BE and BQ sublineages (BE and BQ are sublineages of BA.5 [Fig. 2C]). The N460K mutation has arisen within BA.2 and BA.5 sublineages and XBB recombinants (Fig. 2D). The BA.2 sublineages utilize an AAG codon, whereas the BA.5 lineages utilize an AAA codon. The F486S mutation is slowly becoming more abundant and is seen in BA.2 sublineages and XBB recombinants (Fig. 2E). The F486V mutation arose earliest and differentiates BA.4 and BA.5 lineages from BA.2 lineages (Fig. 2F). Taken together, these mutations are becoming more abundant in circulating SARS-CoV-2 lineages and are arising in multiple independent lineages.

### Digital PCR assays to detect immune evasion-associated SNPs in the spike protein receptor binding domain.

**FIG 3:**
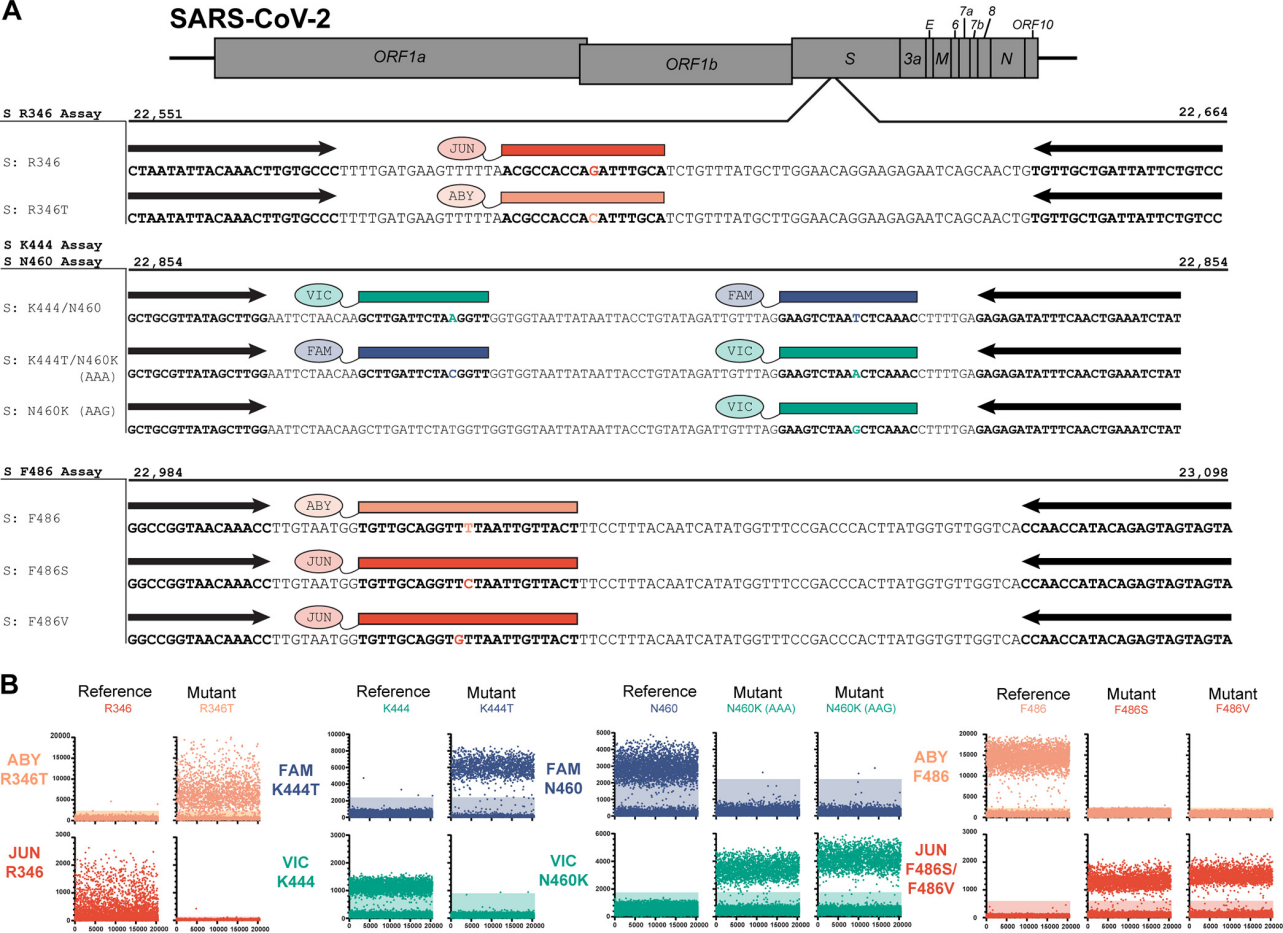
Digital PCR assay for the detection of mutations associated with therapeutic monoclonal antibody evasion. (A) Schematic showing annealing locations of primers (black arrows) and probes (colored boxes) on the SARS-CoV-2 genome. (B) Representative fluorescence intensities of dPCR microchambers for synthetic DNA constructs. Positive microchambers are those exceeding fluorescence thresholds (shaded regions).

Digital PCR oligonucleotides were designed to interrogate the presence and absence of mutations associated with immune evasion at the four genomic loci in the spike RBD region. Each mutation and reference pair contained a different fluorescent dye, and the mutation probes used a different fluorescent dye between residue locations (Fig. 3A). Mutation probes for sites containing two mutations (i.e., N460K [AAA] and N460K [AAG], F486S, and F486V) use the same fluorescent dye for each mutation. The R346 and F486 probe sets use unique, independent amplicons, whereas the K444 and N460 probe sets share a single common amplicon.

In order to determine the effectiveness of the dPCR probes to discriminate the identity of each SNP, reactions containing the probe homologous to the mutation of interest and a probe homologous to the reference sequence were assayed in a single reaction vessel. Each probe pair was assayed using synthetically produced DNA templates containing the mutation nucleotide sequence or reference nucleotide sequence. For each reaction, each probe displayed a higher fluorescence signal to its homologous template than to a template containing a mismatching nucleotide (Fig. 3B). Positive FAM fluorescence was observed from assays with templates containing N460 or K444T residues. Positive VIC fluorescence was observed from assays with templates containing N460K or K444 residues. Positive ABY fluorescence was observed from assays with templates containing R346T or F486 residues. Positive JUN fluorescence was observed from assays using templates containing R346, F486S, or F486V templates. Together, this demonstrates that each probe can correctly discriminate templates differing by only a single SNP within the probe region.

**FIG 4:**
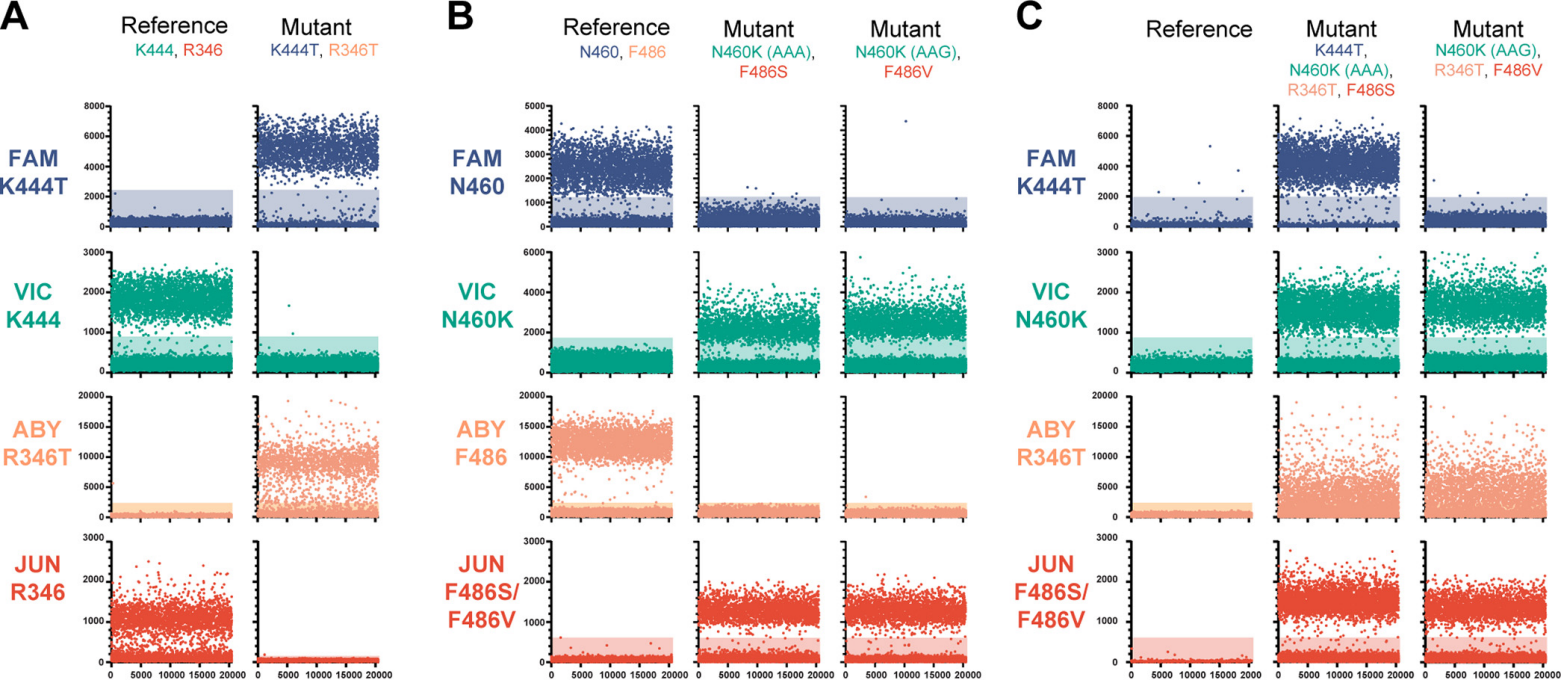
Representative microchamber fluorescence intensities for synthetic DNA constructs from multiplexed dPCR assays. (A) R346 and K444 combination assay. (B) N460 and F486 combination assay. (C) Combination assay using R346T, K444T, N460K (AAA and AAG codons), F486S, and F486V mutation probes. Positive microchambers are those exceeding fluorescence thresholds (shaded regions).

The primers and probes from two assays were combined to assay the identity of two SNPs from a single template. For each reaction, two amplicons were produced, with each amplicon containing only a single substitution of interest. When assayed on synthetic DNA constructs, each probe in the assays displayed higher fluorescence to its homologous template than to a mismatching template. The R346 and K444 assays were combined into a single dPCR assay to interrogate sequence identity at those positions simultaneously (Fig. 4A). On the template containing reference sequences, positive fluorescence was observed in VIC and JUN channels. A positive response was observed in FAM and ABY channels for a template containing K444T and R346T mutations, respectively.

The N460 and F486 assays were also combined into a single assay (Fig. 4B). The reaction mixture contained a labeled probe for each reference sequence, a VIC-labeled probe for each N460K codon (AAG and AAA), and a JUN-labeled probe for each F486 mutation (F486S and F486V). For the reference sequence template, positive fluorescence responses were observed in the FAM and ABY channels, and for both mutation templates, positive responses were observed in the VIC and JUN channels. An equivalent positive response was observed for the N460K AAG and AAA codon probes, as well as between the F486S and F486V probes, for their respective templates.

The presence or absence of mutations at 4 SNP sites was able to be resolved in a single dPCR reaction by using the mutation-specific probes containing a different fluorescent dye for each locus. Three amplicons were produced in each reaction, with the K444 and N460 probes sharing an amplicon. For each probe, fluorescence values were higher for its homologous template than for its mismatching template (Fig. 4C). No positive response from any channel was observed for a template containing the reference sequence at each position. Positive responses in the VIC, ABY, and JUN channels were observed on a template containing N460K (AAG), R346T, and F486V mutations. Positive responses in all four channels were observed on a template containing the K444T, N460K (AAA), R346T, and F486S mutations. The number of positive microchambers were similar between templates and probes except for the R346T probe having a lower positive chamber count. These results demonstrate the versatility of dPCR assays to report the identity of multiple nucleotide sequences across a template.

### A multiplexed dPCR assay detects SARS-CoV-2 immune escape mutations in saliva samples.

In order to test the effectiveness of these multiplexed RT-dPCR assays on clinical samples, we performed dPCR and Illumina sequencing assays on human saliva samples. Saliva samples containing SARS-CoV-2 from 8 lineages, previously identified by Illumina whole-genome sequencing, were used to evaluate the specificity of the assay to discriminate among clinical specimens with various mutation profiles (Table 1). For each lineage, samples were examined using an assay where the R346 and K444 assays were combined, an assay where the N460 and F486 assays were combined, and an assay where all 6 mutation-specific probes were used.

**TABLE 1:** Sample and lineage counts for saliva samples used for each spike RBD dPCR assay

Lineage	Total no. of unique samples^*a*^	No. of samples tested on R346 and K444^*b*^	No. of samples tested on N460K, F486S, and F486V^*c*^	No. of samples tested on R346T, K444T, N460K, F486S, and F486V^*d*^
BA.2	10	3	3	10
BA.2.75.2	10	5	3	9
BA.5	10	3	3	10
BF.7	14	4	4	13
BM.1.1	1	1	1	1
BN.1	7	2	3	6
BQ.1	9	5	3	9
BQ.1.1	12	3	3	11
XBB	8	5	4	5
	81	31	27	74

a Total sample count does not equal the sum of samples from all tests due to samples being used on multiple assays.

b Also see Data Set S3 in the supplemental material.

c Also see Data Set S4.

d Also see Data Set S5.

**FIG 5:**
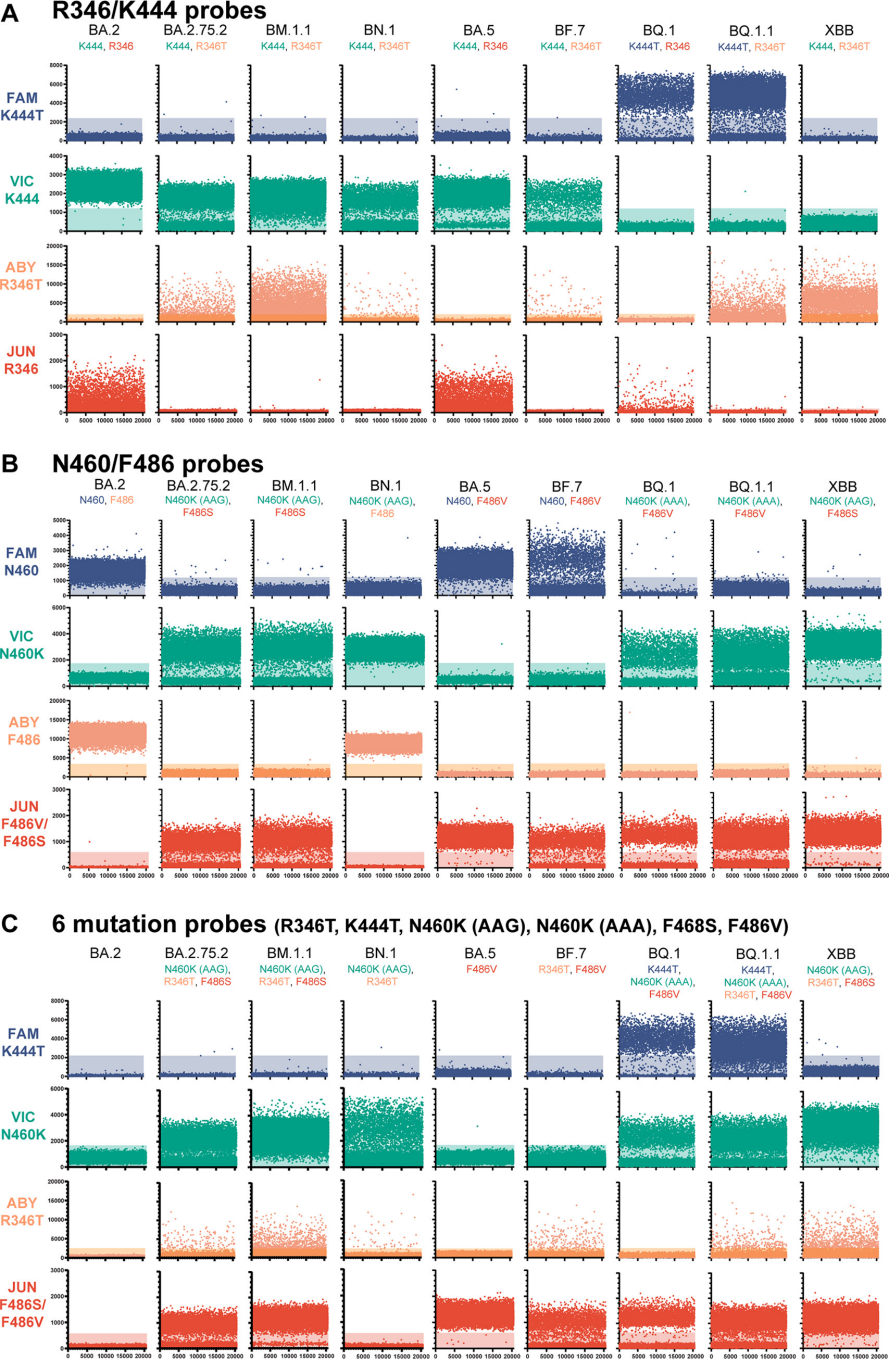
Representative microchamber fluorescence intensities for clinical saliva specimens from multiplexed dPCR assays. (A) R346 and K444 combination assay. (B) N460 and F486 combination assay. (C) Combination assay using R346T, K444T, N460K (AAA and AAG codons), F486S, and F486V mutation probes. Positive microchambers are those exceeding fluorescence thresholds (shaded regions).

The combined R346 and K444 assay was able to correctly discriminate between the mutation and reference sequence at each locus (Fig. 5A; Table 2). FAM fluorescence was observed in the BQ.1 and BQ.1.1 lineages, which contain the K444T mutation. Positive VIC fluorescence was seen in samples from the BA.2, BA.2.75.2, BM.1.1, BN.1, BA.5, and BF.7 lineages, consistent with the presence of the reference sequence at the K444 locus, while the XBB lineage contains the reference amino acid at K444 and V445P mutation. The V445P substitution is not present in the reference-specific probe and causes failure at this position. A positive ABY response was seen in the BA.2.75.2, BM.1.1, BN.1, BF.7, BQ.1.1, and XBB lineages, which contain the R346 mutation. Finally, JUN fluorescence properly identified the presence of the reference sequence at R346 in the BA.2, BA.5, and BQ.1 lineages. A reduced positivity response of the R346 probes was also observed on the saliva samples, as seen with the synthetic DNA constructs. Thirty-two total samples were tested on the combined assay and gave results consistent with their Illumina whole-genome sequencing determination (Data Set S3).

**TABLE 2:** Mutation composition of spike RBD mutations for SARS-CoV-2 sublineages used in this study^*a*^

Lineage	R346	K444	N460	F486
BA.2	Ref	Ref	Ref	Ref
BA.2.75.1	T	Ref	K (AAG)	S
BM.1.1	T	Ref	K (AAG)	S
BN.1	T	Ref	K (AAG)	Ref
BA.5	Ref	Ref	Ref	V
BF.7	T	Ref	Ref	V
BQ.1	Ref	T	K (AAA)	V
BQ.1.1	T	T	K (AAA)	V
XBB	T	Ref	K (AAG)	S

a The N460 mutation lists codon nucleotides in parentheses. Ref, reference residue.

The sequence composition of saliva samples was correctly identified using the N460 and F486 combined assays (Fig. 5B). Positive FAM fluorescence was observed in the BA.2, BA.5, and BF.7 lineages, which contain the reference nucleotide sequence at N460. Positive VIC fluorescence was observed in the BA.2.75.2, BM.1.1, BN.1, BQ.1, BQ.1.1, and XBB lineages, consistent with the presence of the N460K mutation in these lineages. Positive ABY fluorescence was observed in the BA.2 and BA.5 lineages, which have the reference nucleotide sequence at F486. Positive JUN fluorescence was observed in BA.2.75.2, BM.1.1, BA.5, BF.7, BQ.1, BQ.1.1, and XBB lineages, which contain either the F486S or F486V mutations. Twenty-eight total samples were tested on the combined assay and gave results consistent with their Illumina whole-genome sequencing determination (Data Set S4).

An assay comprised of the 6 mutation-specific probes was also verified on saliva samples and gave appropriate responses to mutation composition (Fig. 5C). Positive FAM fluorescence was observed in the BQ.1 and BQ.1.1 lineages, consistent with the presence of the K444T mutation. Positive VIC fluorescence was observed in the BA.2.75.2, BM.1.1, BN.1, BQ.1, BQ.1.1, and XBB lineages, consistent with the presence of either N460K mutation (AAG or AAA codon). Positive ABY fluorescence was observed in the BA.2.75.2, BM.1.1, BN.1, BF.7, BQ.1.1, and XBB lineages, consistent with the presence of the R346T mutation. Positive JUN fluorescence was observed in the BA.2.75.2, BM.1.1, BA.5, BF.7, BQ.1, BQ.1.1, and XBB lineages, consistent with the presence of either the F486S or F486V mutations. No positive fluorescence response was observed in the BA.2 lineage, which contains none of the screened mutations. As in the R346-K444 combination assay, the XBB lineage failed to display FAM fluorescence due to the absence of the K444T mutation and the presence of the V445P mutation. We tested this assay on 75 total saliva samples, and all samples displayed results consistent with their respective Illumina whole-genome sequencing lineage designation (Data Set S5). These assays illustrate that dPCR can discern immunologically relevant SARS-CoV-2 mutations from saliva specimens and could be used to guide therapeutic treatment.

## DISCUSSION

In this study, we demonstrated the robustness of using digital PCR in genotyping SARS-CoV-2-positive saliva samples for two use cases, lineage classification and therapeutic MAb resistance. We showed that using a mixture of probes with a common fluorescent dye can be used to identify mutants when multiple nucleotide substitutions are at one region of interest (N460 and F486 assays) (Fig. 3B). This is particularly useful when an amino acid is encoded by multiple codons or multiple amino acids are of interest. We also demonstrated that detection is not compromised when multiple probes bind to different locations on a single amplicon (N460 and F486 assay) (Fig. 4C). This property is beneficial when interrogating multiple mutations within a single domain of a protein (e.g., the RBD of the S gene) where creating multiple amplicons could be difficult due to primer constraints. Finally, we demonstrated that these assays can be performed on nucleic acids extracted from saliva samples to detect the presence of specific SARS-CoV-2 mutations.

The R346, K444, N460, and F486 residues are locations of key mutations important in neutralization escape, enhanced fusogenicity, and enhanced S protein processing. Structural modeling suggests that R346T and K444T appear to disrupt salt bridge formation between S protein and class III monoclonal antibodies (e.g., cilgavimab), lowering effectiveness (16, 24). The N460K mutation has been shown to enhance S protein processing and is predicted to facilitate a salt bridge and hydrogen bond formation between spike and human ACE2, enhancing cell fusion (25). The F486V mutation aided in evading serum neutralization by early BA.5 lineages, but more recent lineages containing the F486S mutation are even more resistant to neutralization (12). Beyond the mutations examined here, additional mutations at these loci have been implicated in immune escape and are rising in frequency (15).

There are limitations to the use of dPCR for SNP identification. Since dPCR relies on sequence homology between probes, primers, and template nucleic acids, it is susceptible to failure as SARS-CoV-2 evolves mutations. There have been numerous reports of SARS-CoV-2 mutations that cause failure on diagnostic RT-PCR assays (26–28). Mutations in other nucleotides of the probe or primer binding regions will result in failed amplification, as observed with the K444 reference probe against the XBB lineage (Fig. 5A), and could result in sequence misclassification. Further misclassification could arise in the case of coinfections with more than one SARS-CoV-2 sublineage. Either lineage having the mutation of interest would result in a positive result by its respective fluorescent probe assay. The assay results could be interpreted as a single sublineage containing all, even conflicting, mutations.

Furthermore, caution must be exercised in interpreting negative results of reactions containing only a single probe for each SNP of interest (i.e., 6-mutant probe, 4-SNP assay [Fig. 5C]). Results from lineages containing no mutations of interest, such as BA.2, are indistinguishable from failed assays and samples with concentrations below the limit of detection. This limitation could be overcome by using a universally binding probe that would always provide a fluorescent response, but doing so would reduce the total number of SNP sites being interrogated in a single reaction. For currently circulating Omicron variants, the Orf1ab 3395 NED probe could be used for this purpose and is compatible with the spike RBD primers (see Fig. S3 in the supplemental material).

This study aims to provide proof of concept for the use of dPCR to aid in personalized patient diagnostics, but transitioning a dPCR assay from research-oriented applications to a diagnostic assay will require further testing of any primer/probe concentration against other pathogens and determine their limits of detection, reproducibility, and sensitivity. In this report, 56 samples failed to receive a positive result from any probe on the Orf1ab 3395 lineage-discriminating assay. Since genome analysis revealed no sequence mismatches in primer and probe regions, failure may be due to degradation of RNA occurring after the time of initial collection. In this study, samples were tested for qRT-PCR, then Illumina sequencing, followed by dPCR assays. Each sample may have been subjected to 1 to 4 freeze-thaw cycles before dPCR testing due to sample handling and management. Each freeze-thaw cycle could result in RNA degradation.

In this study, assays interrogating the R346 mutation often had an indistinct separation between positive and negative fluorescence values and fewer positive wells than other mutation loci (see Fig. 5). Adjustment of the dPCR assay temperature did not improve assay performance. A redesign of primers and probes did result in improvement in synthetic DNAs but only partial improvement in saliva samples (Fig. S4). On saliva samples, the separation between fluorescence values of positive and negative wells was increased, but the resulting number of positive wells was still 3 to 5 times lower than the count for other probes. This mutation may lie at a problematic location of the genome caused by secondary structure formation or other confounding characteristics.

As new variants emerge, the effectiveness of current antibody therapies is at risk. At their emergence, the initial Omicron variants were less sensitive to antibody treatments bamlanivimab and etesevimab (joint administration) and REGEN-COV (casirivimab and imdevimab), and their use was curtailed (29). More recent variants have caused the recall of emergency use authorization for bebtelovimab, leaving paxlovid (nirmatrelivir-ritonavir) and remdesivir as preferred treatment options (30). These treatments are also at risk, as *in vitro* analysis using a vesicular stomatitis virus system has identified that Y54C, G138S, L167F, Q192R, A194S, and F305L mutations in nsp5 (3-cymotrypsin-like protease) could result in reduced paxlovid efficacy (31). As demonstrated in this study, a similar dPCR approach could be used to determine resistance to paxlovid. Due to the very rapid development of resistance mutations in SARS-CoV-2 (32), there is an increased need for versatile assays to guide patient treatment and extend the use of life-saving therapeutics. The use of digital PCR to clarify active infection (33), determine SARS-CoV-2 lineages, and pinpoint therapeutically relevant mutations make it a well-suited technology for use in personalized diagnostics.

## MATERIALS AND METHODS

### Saliva specimens and diagnostic testing.

This study was approved by Arizona State University Institutional Review Board. This study involved analyses of 677 saliva specimens submitted for SARS-CoV-2 testing at ASU Biodesign Clinical Testing Laboratory (ABCTL) from 16 November 2021 to 10 December 2022. Saliva samples were independently collected by participants in 2-mL collection vials, registered, and deposited at drop-off locations. RNA was extracted from 250 μL of saliva specimen within 33 h of sample receipt using the KingFisher Flex (Thermo Scientific), following the manufacturer’s guidelines. Diagnostic testing was performed using TaqPath coronavirus disease 2019 (COVID-19) Combo kit assay (Applied Biosystems, USA) following the manufacturer’s guidelines.

### Digital PCR.

DNA constructs (primers, probes, template constructs) were ordered from either Integrated DNA Technologies (Coralville, IA, USA) or Applied Biosystems (Waltham, MA, USA) (nucleotide sequences can be found in Tables S1, S2, and S3 in the supplemental material). The mismatch frequency at each nucleotide position was calculated for all primers and probes against all genome sequences in the GISAID database. We found that probes had low mismatch incidence outside the codon encoding the mutation of interest (Fig. S1). Primers also had a low mismatch frequency; however, the F486 primers require the presence of the S477N and T478K SNPs for amplicon generation.

The reaction mixtures for the *orf1ab* and spike lineage discriminating assays were comprised of 1× Absolute Q 1-step RT-dPCR master mix (Applied Biosystems, Waltham, MA), 400 nM of each primer, 200 nM of each fluorescent probe, and 4 μL template; water was used to bring the final volume to 9 μL. Synthetic DNAs were assayed at a concentration of 10^3^ copies/μL. Saliva specimens with threshold cycle (*C_T_*) values less than 19 when assayed using TaqPath COVID-19 Combo kit assay were diluted 1:2 before loading.

The reaction mixtures for the spike RBD mutation assays were comprised of 1× Absolute Q 1-step RT-dPCR master mix, 450 nM of each primer, 280 nM of each fluorescent probe, and 5.5 μL template; water was used to bring the final volume to 9 μL. Synthetic DNAs were assayed at a concentration of 10^3^ copies/μL.

Samples were loaded in QuantStudio Absolute Q MAP16 (Applied Biosystems) and overlaid with 15 μL Absolute Q isolation buffer (Applied Biosystems), and the digital PCRs were performed on the Absolute Q dPCR system (Applied Biosystems). For the *orf1ab* and spike lineage determination assays, cycling conditions were 10 min at 50°C, 5 min activation at 95°C, and 40 cycles of 2 s at 95°C and 15 s at 54°C. For the spike RBD assay determination assays, cycling conditions were 10 min at 50°C, 5 min activation at 95°C, and 40 cycles of 2 s at 95°C and 25 s at 58°C.

Fluorescence intensities and positive chamber counts were analyzed using QuantStudio Absolute Q Digital PCR software version 6.2.0 (Applied Biosystems). For each probe, fluorescence thresholds were set to exclude fluorescence values obtained from DNA templates containing non-cDNA sequences at the binding site. For the lineage-determining probes (Orf1ab_3395), the noncomplementary sequences were each other lineages being interrogated. For the spike RBD assays (R346, K444, N460K, F486S, and F486V), noncomplementary sequences were the Wuhan-Hu-1 sequence and the SNP sequences for any other mutations interrogated at that site. For all assays, a 9-positive-well criterion for sample positivity was set to account for a small amount of nonspecific amplification observed in some samples. Positive well counts for clinical specimens can be found in Data Sets S1 to S5.

### SARS-CoV-2 genome sequencing.

NGS library preparation for saliva samples was performed using the COVIDSeq test (Illumina, San Diego, CA, USA) with ARTICv4 and ARTICv4.1 primer sets (34). Libraries were sequenced on the Illumina NextSeq2000 instrument using 2 × 109 paired-end reads. Sequencing read adapter sequences were trimmed using Trim Galore aligned to the Wuhan-Hu-1 reference genome (GenBank accession no. MN908947) using the Burrows-Wheeler aligner, BWA-MEM version 0.7.17-r1188 (35), and had their primer sequences trimmed using iVAR version 1.3.1 (36). Lineage calling for community- and hospital-derived sequencing data was performed with pangolin software (37), with its assignment and designation libraries up to date at the time of analysis. Sequence quality was validated and annotated using VADR version 1.4 (38).

### Phylogenetic analysis.

Phylogenies were generated using Nextstrain CLI version 5.0.1 (39) and associated Augur Auspice pipeline with an input data set based on the Nextstrain ncov GISAID reference data set accessed on 16 November 2022. Duplicate lineages were removed, and the set was supplemented with lineages of interest in which assay-targeted mutations were found to be present in at least 75% of sequences. The Nextstrain ncov default build was utilized, with a minimal config file specifying the input data set and Wuhan/Hu-1/2019 as the root. The filter settings in the default parameters file were modified by setting the skip_diagnostics flag to true.

### Global mutation proportion analysis.

Global mutation proportion data were downloaded from covSPECTRUM (40) with simple queries for S:R346T, S:K444T, S:N460K, S:F486S, and S:F486V. Absolute counts were downloaded in addition to proportion data for S:N460K. An advanced query was performed to obtain absolute counts for each codon (AAA, AAG) present in S:N460K sequences. Data were plotted from 3 March 2022 until 6 November 2022. For the plotted data range, the difference in the top 95% confidence interval (CI) and bottom 95% CI never reaches greater than 5%.

### Availability of data and materials.

Genome sequences have been deposited to the GISAID repository. Scripts for data curation can be found on the Lim lab GitHub (https://github.com/ASU-Lim-Lab/Absolute-Q).
