# Supplementary material for: Digital PCR Discriminates between SARS-CoV-2 Omicron Variants and Immune Escape Mutations
Source: Microbiol Spectr. 2023 Jun 12;11(4):e05258-22. doi: 10.1128/spectrum.05258-22 (PMC10434287; doi:10.1128/spectrum.05258-22)
Supplement: Supplemental file 1 — Supplemental material. Download spectrum.05258-22-s0001.pdf, PDF file, 1.1 MB [file spectrum.05258-22-s0001.pdf]

Supplemental material

Table S1: Digital PCR probe sequences and modifications

Probe	Sequence	5’ Fluorescent dye	3’ modification
Orf1ab3395_BA1_FAM	CTATGAGGCACAATT	FAM	MGBNFQ
Orf1ab3395_Delta_VIC	CTATGAGGCCCAATT	VIC	MGBNFQ
Orf1ab3395_BA2_NED	CTATGAGACACAATT	NED	MGBNFQ
S143_BA.1_FAM	CATTTTTGGACCACAAAAA	FAM	MGBNFQ
S143_DeltaBA2_VIC	GGATGTTTATTACCACA	VIC	MGBNFQ
R346_JUN	ACGCCACCAGATTTGCA	JUN	QSY
R346T_ABY	ACGCCACCACATTTGCA	ABY	QSY
K444_VIC	GCTTGATTCTAAGGTT	VIC	MGBNFQ
K444T_FAM	GCTTGATTCTACGGTT	FAM	MGBNFQ
N460_FAM	GAAGTCTAATCTCAACC	FAM	MGBNFQ
N460Kaag_VIC	GAAGTCTAAACTCAAAC	VIC	MGBNFQ
N460Kaaa_VIC	GAAGTCTAAGCTCAAAC	VIC	MGBNFQ
F486_ABY	TGTTGCAGGTTTTAATTGTTACT	ABY	QSY
F486S_JUN	TGTTGCAGGTTCTAATTGTTACT	JUN	QSY
F486V_JUN	TGTTGCAGGTGTTAATTGTTACT	JUN	QSY
R346T_NED (Alt)	AACGCCACCACATTTGCATC	NED	MGBNFQ
R346_JUN (Alt)	AACGCCACCAGATTTGCATC	JUN	QSY

Table S2: Digital PCR primer sequences

Primer	Sequence	Notes
Orf1ab3395_fwd	TGTTAGCTTGTTACAATGGTTCACC	
Orf1ab3395_rev	CCACATGAACCATTAAGGAATGAACC	
S143_fwd	GCTACTAATGTTGTTATTAAAGTCTGTGAATTTC	Supplemental figure
S143_rev	GTGCAATTATTCGCACTAGAATAAACTC	Supplemental figure
R346_fwd	CTAATATTACAAACTTGTGCCC	
R346_rev	GGACAGAATAATCAGCAACA	
K444_N460_fwd	GCTGCGTTATAGCTTGG	
K444_N460_rev	ATAGATTTCAGTTGAAATATCTCTC	
F486_fwd	GGCCGGTAACAAACC	
F486_rev	TACTACTACTCTGTATGGTTGG	
R346_fwd (Alt)	GATTTCCTAATATTACAAACTTGTGC	Supplemental figure
R346_rev (Alt)	AGGACAGAATAATCAGCAACACA	Supplemental figure

Table S3: Digital PCR synthetic DNA templates

Name	Sequence	Mutations from Wuhan1 (probe-specific mutations in bold)
Orf1ab3395_Delta	CGCTGTAATACGACTCACTATAGGGTTAAGGTTG ATACAGCCAATCCTAAGACACCTAAGTATAAGTT TGTTCGCATTCAACCAGGACAGACTTTTTCAGTG TTAGCTTGTTACAATGGTTCACCATCTGGTGTTTA CCAATGTGCTATGAGGCCCAATTTCACTATTAAG GGTTCATTCCTTAATGGTTCATGTGGTAGTGTTG GTTTTAACATAGATTATGACTGTGTCTCTTTTTGT TACATGCACCATATGGAATTACCAACTGGAGTTC ATGCTGGCACAGACTTA	None
Orf1ab3395_BA1	CGCTGTAATACGACTCACTATAGGGTTAAGGTTG ATACAGCCAATCCTAAGACACCTAAGTATAAGTT TGTTCGCATTCAACCAGGACAGACTTTTTCAGTG TTAGCTTGTTACAATGGTTCACCATCTGGTGTTTA CCAATGTGCTATGAGGCACAATTTCACTATTAAG GGTTCATTCCTTAATGGTTCATGTGGTAGTGTTG GTTTTAACATAGATTATGACTGTGTCTcTTTTTGTT ACATGCACCATATGGAATTACCAACTGGAGTTCA TGCTGGCACAGACTTA	**P3395H**
Orf1ab3395_BA2	CGCTGTAATACGACTCACTATAGGGTTAAGGTTG ATACAGCCAATCCTAAGACACCTAAGTATAAGTT TGTTCGCATTCAACCAGGACAGACTTTTTCAGTG TTAGCTTGTTACAATGGTTCACCATCTGGTGTTTA CCAATGTGCTATGAGACACAATTTCACTATTAAG GGTTCATTCCTTAATGGTTCATGTGGTAGTGTTG GTTTTAACATAGATTATGACTGTGTCTCTTTTTGT TACATGCACCATATGGAATTACCAACTGGAGTTC ATGCTGGCACAGACTTA	**a10447g** (Synonymous R3394), **P3395H**
S143_Delta/BA.2	CGCTGTAATACGACTCACTATAGGGAGAGGCTG GATTTTTGGTACTACTTTAGATTCGAAGACCCAG TCCCTACTTATTGTTAATAACGCTACTAATGTTGT TATTAAAGTCTGTGAATTTCAATTTTGTAATGATC CATTTTTGGATGTTTATTACCACAAAAACAACAAA AGTTGGATGGAAAGTGGAGTTTATTCTAGTGCGA ATAATTGCACTTTTGAATATGTCTCTCAGCCTTTT CTTATGGACCTTGAAGGAAAACAGGGTAATTTCA AAAA	
S143_BA1	CGCTGTAATACGACTCACTATAGGGTAATAAGAG GCTGGATTTTTGGTACTACTTTAGATTCGAAGAC CCAGTCCCTACTTATTGTTAATAACGCTACTAATG TTGTTATTAAAGTCTGTGAATTTCAATTTTGTAAT GATCCATTTTTGGACCACAAAAACAACAAAAGTT GGATGGAAAGTGAGTTCAGAGTTTATTCTAGTGC GAATAATTGCACTTTTGAATATGTCTCTCAGCCTT TTCTTATGGACCTTGAAGGAAAACAGGGTAATTT CAAAAATCTT	**G142D**, **del143-145**

RBD_Ref-like	TAATACGACTCACTATAGGGAGATTAGATTTCCT<br>AATATTACAAACTTGTGCCCTTTTGATGAAGTTTT<br>TAACGCCACCAGATTTGCATCTGTTTATGCTTGG<br>AACAGGAAGAGAATCAGCAACTGTGTTGCTGATT<br>ATTCTGTCCTATATAATTTCGCACCATTTTTCGCT<br>TTTAAGTGTTATGGAGTGTCTCCTACTAAATTAAA<br>TGATCTCTGCTTTACTAATGTCTATGCAGATTCAT<br>TTGTAATTAGAGGTAATGAAGTCAGCCAAATCGC<br>TCCAGGGCAAACTGGAAATATTGCTGATTATAAT<br>TATAAATTACCAGATGATTTTACAGGCTGCGTTAT<br>AGCTTGGAATTCTAACAAGCTTGATTCTAAGGTT<br>GGTGGTAATTATAATTACCTGTATAGATTGTTTAG<br>GAAGTCTAATCTCAAACCTTTTGAGAGAGATATTT<br>CAACTGAAATCTATCAGGCCGGTAACAAACCTTG<br>TAATGGTGTTGCAGGTTTTAATTGTTACTTTCCTT<br>TACAATCATATGGTTTCCGACCCACTTATGGTGT<br>TGGTCACCAACCATACAGAGTAGTAGTACTTTCT<br>TTT	G339D, S371F, S373P, S375F, T376A, D405N, R408S, K417N, N440K, S477N, T478K, E484A, Q498R, N501Y, Y505H
RBD_Mutant1	TAATACGACTCACTATAGGGAGATTAGATTTCCT<br>AATATTACAAACTTGTGCCCTTTTGATGAAGTTTT<br>TAACGCCACCACATTTGCATCTGTTTATGCTTGG<br>AACAGGAAGAGAATCAGCAACTGTGTTGCTGATT<br>ATTCTGTCCTATATAATTTCGCACCATTTTTCGCT<br>TTTAAGTGTTATGGAGTGTCTCCTACTAAATTAAA<br>TGATCTCTGCTTTACTAATGTCTATGCAGATTCAT<br>TTGTAATTAGAGGTAATGAAGTCAGCCAAATCGC<br>TCCAGGGCAAACTGGAAATATTGCTGATTATAAT<br>TATAAATTACCAGATGATTTTACAGGCTGCGTTAT<br>AGCTTGGAATTCTAACAAGCTTGATTCTACGGTT<br>GGTGGTAATTATAATTACCGGTATAGATTGTTTAG<br>GAAGTCTAAACTCAAACCTTTTGAGAGAGATATTT<br>CAACTGAAATCTATCAGGCCGGTAACAAACCTTG<br>TAATGGTGTTGCAGGTTCTAATTGTTACTTTCCTT<br>TACAATCATATGGTTTCCGACCCACTTATGGTGT<br>TGGTCACCAACCATACAGAGTAGTAGTACTTTCT<br>TTT	G339D, **R346T**, S371F, S373P, S375F, T376A, D405N, R408S, K417N, N440K, **K444T**, L452R, **N460K** (aaa codon), S477N, T478K, E484A, **F486S**, Q498R, N501Y, Y505H
RBD_Mutant2	TAATACGACTCACTATAGGGAGATTAGATTTCCT<br>AATATTACAAACTTGTGCCCTTTTGATGAAGTTTT<br>TAACGCCACCACATTTGCATCTGTTTATGCTTGG<br>AACAGGAAGAGAATCAGCAACTGTGTTGCTGATT<br>ATTCTGTCCTATATAATTTCGCACCATTTTTCGCT<br>TTTAAGTGTTATGGAGTGTCTCCTACTAAATTAAA<br>TGATCTCTGCTTTACTAATGTCTATGCAGATTCAT<br>TTGTAATTAGAGGTAATGAAGTCAGCCAAATCGC<br>TCCAGGGCAAACTGGAAATATTGCTGATTATAAT<br>TATAAATTACCAGATGATTTTACAGGCTGCGTTAT<br>AGCTTGGAATTCTAACAAGCTTGATTCTATGGTT<br>GGTGGTAATTATAATTACCGGTATAGATTGTTTAG<br>GAAGTCTAAGCTCAAACCTTTTGAGAGAGATATT<br>TCAACTGAAATCTATCAGGCCGGTAACAAACCTT<br>GTAATGGTGTTGCAGGTGTTAATTGTTACTTTCCT<br>TTACAATCATATGGTTTCCGACCCACTTATGGTG<br>TTGGTCACCAACCATACAGAGTAGTAGTACTTTC<br>TTTT	G339D, **R346T**, S371F, S373P, S375F, T376A, D405N, R408S, K417N, N440K, K444M, L452R, **N460K** (aag codon), S477N, T478K, E484A, **F486V**, Q498R, N501Y, Y505H

Supplemental Figure S1

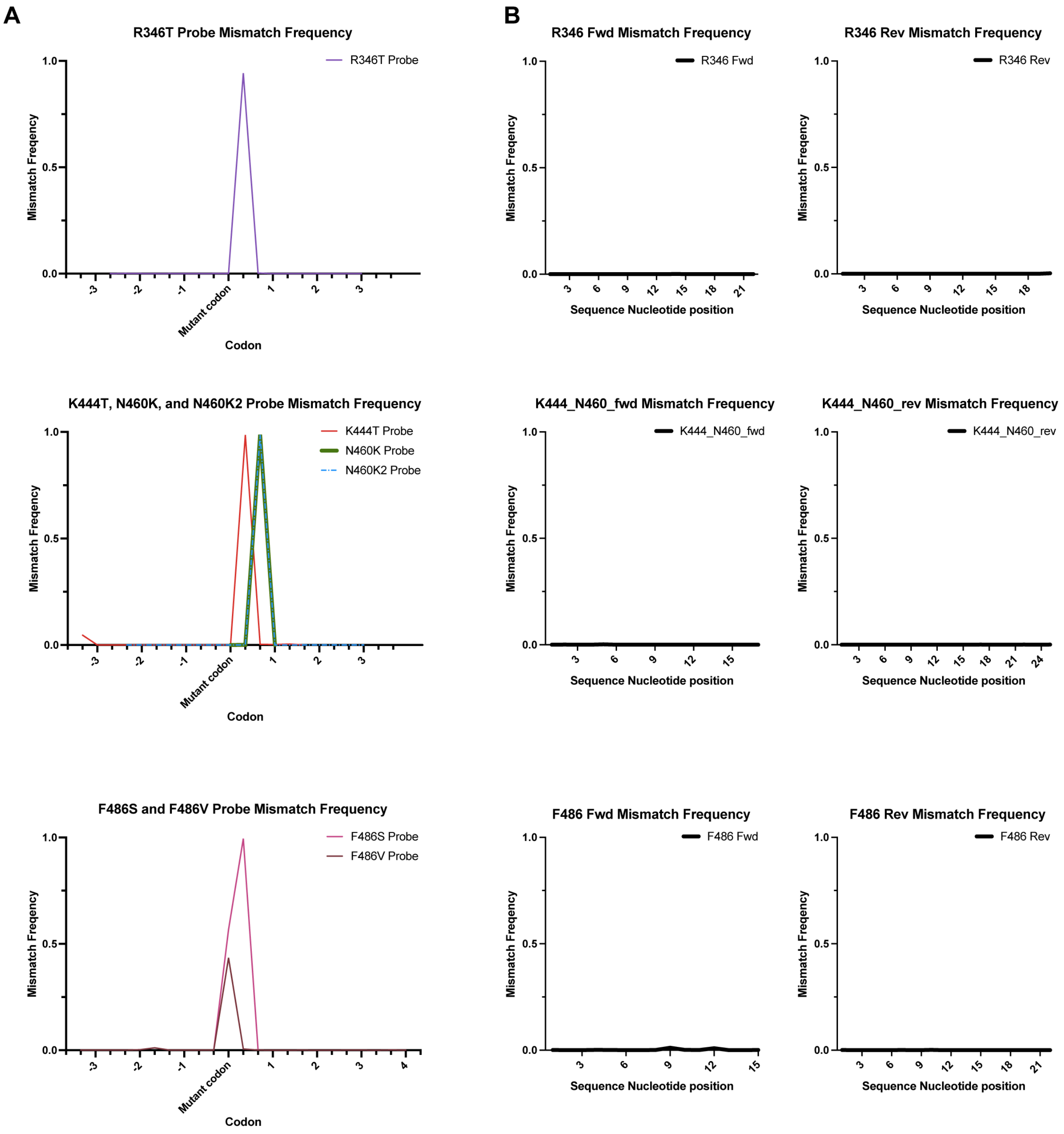

Fig S1: Failure frequency of probes (A) and primers (B) used in the *spike* RBD digital PCR assays.

Supplemental Figure S2

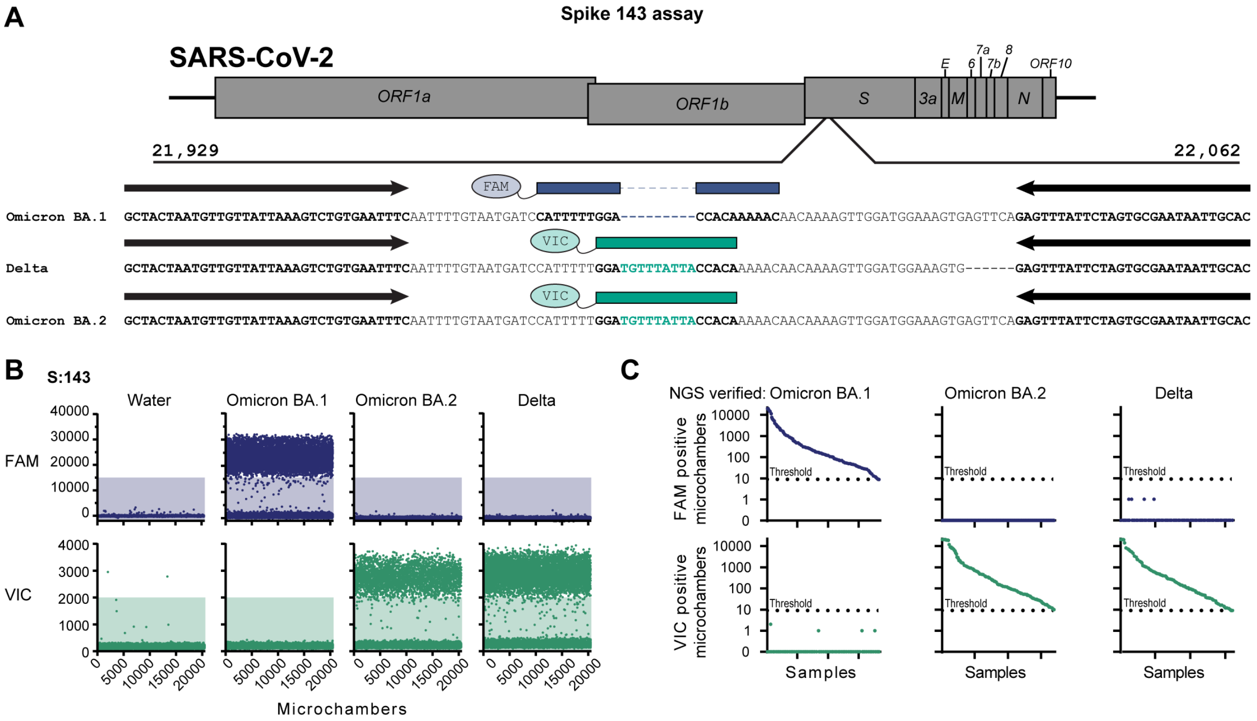

Fig S2: Digital PCR assay for the determination of the Omicron BA.1, and Delta or Omicron BA.2 lineages. (A) Schematic showing annealing locations of primers (black arrows) and probes (colored boxes) on the SARS-CoV-2 genome. (B) Representative fluorescence intensities of dPCR microchambers for synthetic DNA constructs. Positive microchambers are those exceeding fluorescence thresholds (shaded regions). (C) Number of positive microchambers (maximum number of chambers is 20480) resulting from each saliva sample. Samples are grouped by Illumina sequencing lineage determination and sorted by positive microchamber count of the respective lineage-specific probe. Dotted line indicates the positive threshold value (9 microchambers).

Supplemental Figure S3

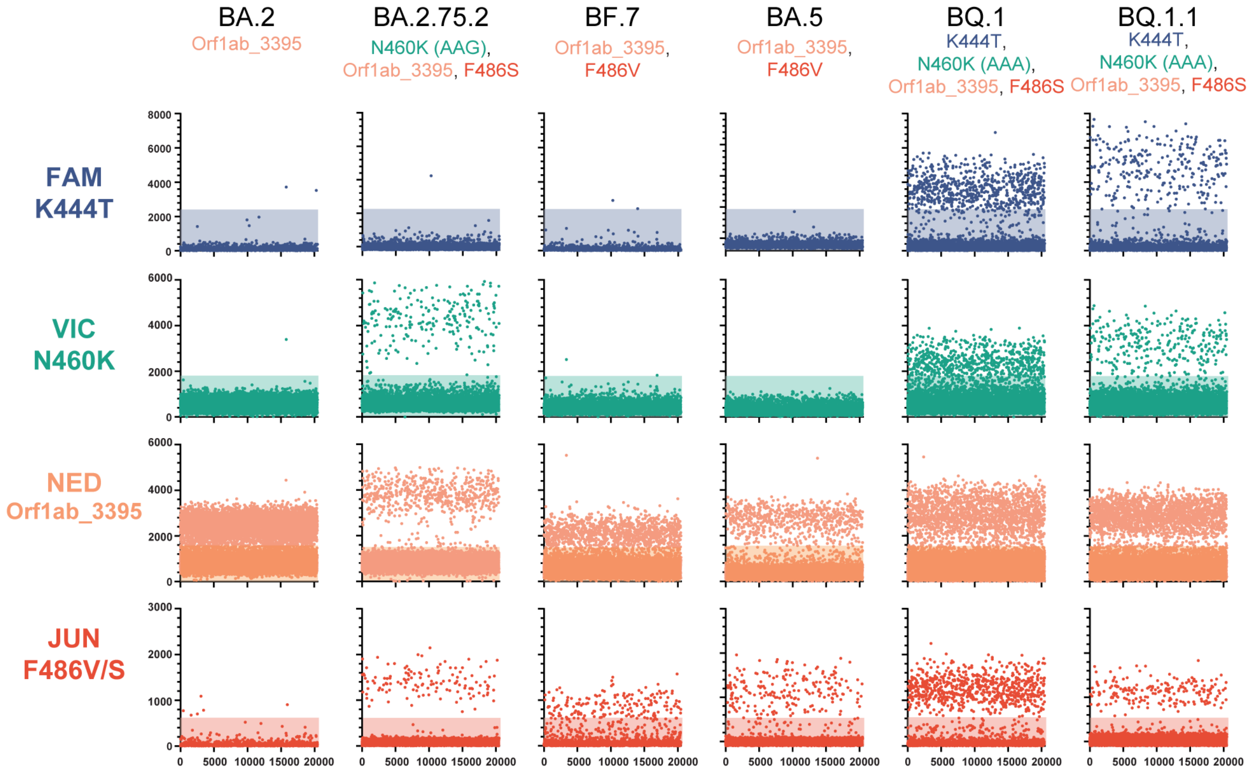

Fig S3: Representative microchamber fluorescence intensities for clinical saliva specimens from multiplexed Orf1ab and S gene dPCR assays. Positive microchambers are those exceeding fluorescence thresholds (shaded regions).

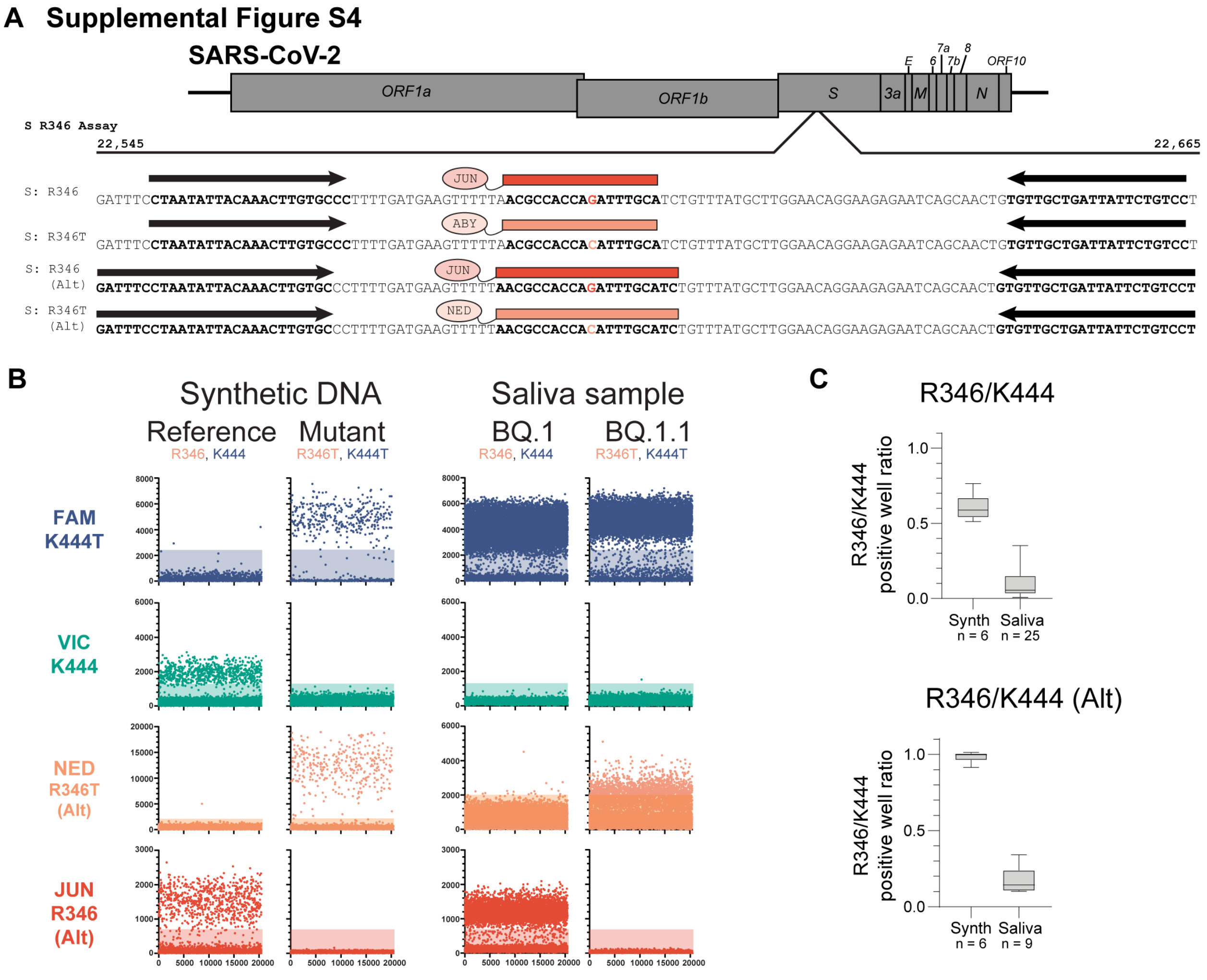

Fig S4: Alternate dPCR primer/probe assay for R346 analysis. (A) Schematic showing annealing locations of primers (black arrows) and probes (colored boxes) on the SARS-CoV-2 genome. (B) Representative microchamber fluorescence intensities for a multiplexed dPCR assays using the alternate R346 primer/probes with the K444 primer/probe set. Template nucleic acid is either synthetic DNA constructs (left) or saliva sample extracts (right). (C) Ratio of the number of positive microchamber wells from R346 probes to the number of positive microchamber wells from K444 probes. Assays were performed using R346 primer/probes (See **Fig 4A**) or R346 alternate set.
